# A plant biostimulant from the seaweed Ascophyllum nodosum (Sealicit) reduces podshatter and yield loss in oilseed rape through modulation of *IND* expression

**DOI:** 10.1038/s41598-019-52958-0

**Published:** 2019-11-12

**Authors:** Łukasz Łangowski, Oscar Goñi, Patrick Quille, Pauline Stephenson, Nicholas Carmody, Ewan Feeney, David Barton, Lars Østergaard, Shane O’Connell

**Affiliations:** 1 Plant Biostimulant Group, Shannon Applied Biotechnology Centre, Institute of Technology Tralee, Clash, Tralee, Co., Kerry, Ireland; 2 Brandon Bioscience, Centrepoint, Tralee, Co., Kerry, Ireland; 3 Department of Crop Genetics, John Innes Centre, Norwich Research Park, Colney, NR4 7UH Norfolk, Norwich, United Kingdom

**Keywords:** Biomaterials, Field trials, Plant development, Seed distribution

## Abstract

The yield of podded crops such as oilseed rape (OSR) is limited by evolutionary adaptations of the plants for more efficient and successful seed dispersal for survival. These plants have evolved dehiscent dry fruits that shatter along a specifically developed junction at carpel margins. A number of strategies such as pod sealants, GMOs and hybrids have been developed to mitigate the impact of pod shatter on crop yield with limited success. Plant biostimulants have been shown to influence plant development. A challenge in plant biostimulant research is elucidating the mechanisms of action. Here we have focused on understanding the effect of an Ascophyllum nodosum based biostimulant (Sealicit) on fruit development and seed dispersal trait in Arabidopsis and OSR at genetic and physiological level. The results indicate that Sealicit is affecting the expression of the major regulator of pod shattering, *INDEHISCENT*, as well as disrupting the auxin minimum. Both factors influence the formation of the dehiscence zone and consequently reduce pod shattering. Unravelling the mode of action of this unique biostimulant provides data to support its effectiveness in reducing pod shatter and highlights its potential for growers to increase seed yield in a number of OSR varieties.

## Introduction

For centuries, the careful selection and crossing of the best performing plants, along with steady improvement of agricultural practices were at the heart of increasing crop productivity. In the twentieth century with the beginning of the Green Revolution, crop production increased rapidly due to the employment of new technologies, high-yielding varieties, chemical fertilisers, agricultural chemicals for crop protection and efficient irrigation^1^. Although agricultural development continues, gains in productivity have plateaued due to abiotic stress, soil degradation, pollution and pressure from pathogens. These factors have made the achievement of a crop’s genetic potential increasingly challenging^2,3^.

Besides the previously mentioned factors, crop yield is also limited by evolutional adaptations of plants for more efficient and successful seed dispersal. These limitations are particularly evident and problematic in the two most important oil crops, namely soybean and OSR, which evolved dehiscent dry fruits, that shatter along a specifically developed junction at carpel margins^4,5^. Recent reports from a number of countries around the globe show that soybean seed loss associated with pod shattering is largely affected by the weather and may vary between 5% to an extreme 100% in susceptible varieties at delayed harvest^6–9^. The second largest oil crop OSR, with worldwide production exceeding 74 million metric ton, records on average in UK 15–25% pod shattering associated seed loss and up to 70% in extreme cases, which translates approximately to $70 million loss in the UK alone^10^. For the entire European production, which consists almost one third of global supply, losses are tenfold higher^11^. Therefore, the mechanism behind shattering and seed dispersal has been closely studied in the model plant *Arabidopsis thaliana* as well as OSR (*Brassica napus*)^12–15^. Both species belong to the Brassicaceae family that evolved dry dehiscent fruits derived from cylindrical carpels that encapsulate developing seeds^5,16^. The lateral parts of the fruits are developed into valves, and the ovules/seed protecting layers are fused to the central replum through just a few cell files. This, so called dehiscence zone or valve margin, is crucial for seed release upon maturity^5,14,17^. Many of the key regulators of fruit development and particularly valve margin development have been identified. *FRUITFUL* (*FUL*) and *REPLUMLESS* (*RPL*) genes specify valve and replum formation^18–21^, partially by restricting the valve margin identity genes *SHATTERPROOF1/2* (*SHP1/2*), *INDEHISCENT* (*IND*) and *ALCATRAZ* (*ALC*)^12,13,22–26^. MADS-box genes *SHP1/2* are on the top of the signalling cascade and their elimination leads to the reduction of the lignified layer and separation layer in the dehiscence zone that ultimately results in a more indehiscent fruit^12^. Acting downstream and in parallel to *SHP1/2*, *IND* and *ALC* valve margin identity. While *ALC* promotes the separation layer development, *IND* regulates the formation of the separation and lignification layers^12,24^. As demonstrated by Liljegren *et al*., 2004^12^, the elimination of *IND* results in no lignified cells throughout the entire dehiscence zone, resulting in extremely shatter-resistant fruits. Next to transcription factors, proper valve margin differentiation and dehiscence zone development is closely related to hormone homeostasis. By regulating AGC3 kinases, IND precisely regulates auxin levels through PIN3-mediated auxin efflux^27,28^. On the contrary, auxin levels regulate *IND* expression leading to indehiscent fruits^27^.

In the last decades the prevention of shattering was mostly tackled by classical breeding and genetic modifications^14^. Currently, the global tendency is to drive productivity by increasing crop yield/quality in an environmentally sustainable manner, which creates an unprecedented opportunity for plant biostimulants to play an important role. Plant biostimulants contain substance(s) and/or micro-organisms whose function when applied to plants or the rhizosphere is to stimulate natural processes to enhance/benefit nutrient uptake, nutrient efficiency, tolerance to abiotic stress, and crop quality^29^. The global biostimulants market is projected by some analysts to reach $4.14 billion by 2025, with Europe projected to be the largest revenue-generating region^30^. Seaweed extracts are prominent in the biostimulant market, representing the fastest growing biostimulant product category^29^. The effects of seaweed extracts, in particular *Ascophyllum nodosum* extracts (ANE), on plants have been reviewed in detail^31,32^. ANE biostimulants have been shown to improve plant vigour, increase root development, enhance chlorophyll synthesis, promote earlier flowering, enhance fruit set and uniformity of fruit, delay senescence and enhance tolerance to abiotic stress^33–42^. However, the role of specifically formulated ANEs in enhancing the quality of pods in oilseed rape (OSR) has not previously been reported. Demonstration of a beneficial effect would highlight the potential for biostimulants in enhancing crop quality with subsequent productivity gains.

In this study, multiple experimental approaches were employed to explore the mode of action (MOA) of a novel ANE biostimulant, Sealicit which was developed utilising a targeted plant signal induction (PSI) approach to formulation development. In multi-annual field trials Sealicit was found to increase the yield of a number of OSR varieties. Observed developmental changes indicated that Sealicit has an impact on key genetic players determining fruit development and potentially seed dispersal. In order to test our hypothesis, that Sealicit reduces pod shatter and increases yield, fruits of Arabidopsis and OSR plants treated with Sealicit were analysed using phenotyping, genetic tools and confocal microscopy. Here we show that thoroughly tested and refined biostimulants can be employed to enhance crop quality by providing desirable traits without interference into the genome or time-consuming classical breeding thereby bringing exciting opportunities for the agriculture of the future.

## Results

### Sealicit affects fruit morphology in *Arabidopsis thaliana*

**Figure 1:**
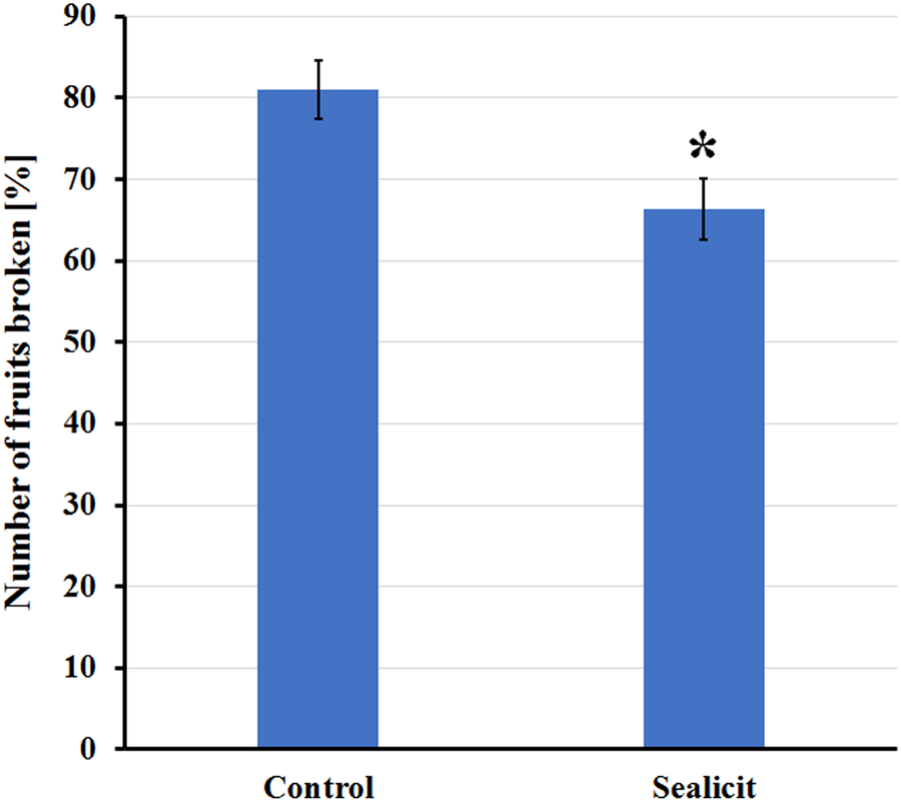
RIT on Arabidopsis fruits representing percentage of fruits broken. Fruit firmness measured by RIT on siliques collected at stage 18. Each bar represents the percentage of open or damaged fruits. The error bars represent SE and means followed by asterisk indicate significant differences between control and the Sealicit treatment based on t-test (*p* ≤ 0.05). Number of analysed samples (n = 40–110).

Biostimulants have been reported to influence plant growth and development, therefore, to gain some insight on the impact of Sealicit on fruit development, we characterized the fruit morphology of *Arabidopsis thaliana*. Siliques at stage 17b, when fruits are still green but fully extended (according to classification of Smyth *et al*.^43^), were analysed with respect to their length and weight. We observed that fruits of treated plants were significantly longer (*p* ≤ 0.001) and heavier (*p* ≤ 0.001), when compared to those from water sprayed controls (Fig. S1A,SB). Then, we tested whether treatments had any effect on seed dispersal. Using the recently developed random impact test (RIT) method^44^, fruit firmness upon exposure to mechanical shaking was evaluated. Due to the differences in fruit size, the experimental setup for Arabidopsis and OSR differed. Our experiments revealed that Arabidopsis plants sprayed with Sealicit showed improved pod shatter resistance (*p* = 0.015) in comparison to the control (Figs 1 and S1C).

### Sealicit affects dehiscence zone development

**Figure 2:**
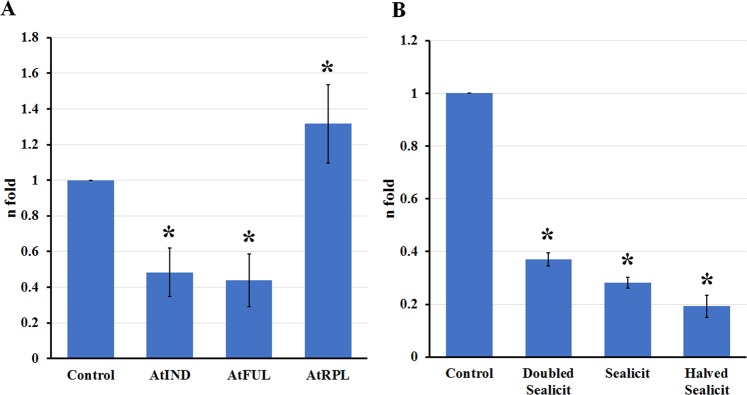
Relative expression of major genes determining fruit development in Arabidopsis. Relative expression of *AtIND*, *AtFUL* and *AtRPL* in Arabidopsis fruits at stage 17b (**A**), and *AtIND* in pods at the same growth stage collected from plants sprayed with different Sealicit concentrations (**B**). Results are expressed as the relative log2 fold-change with respect to the *AtUBC21* gene expression level. The error bars represent SE and means followed by asterisk indicate significant differences between control and the Sealicit treatments based on one-way analysis of variance (ANOVA). The significance level was set at *p* ≤ 0.05 and performed by Holm-Sidak’s test. RNA extractions followed by RTq-PCR experiments were performed in triplicate (n = 3).

**Figure 3:**
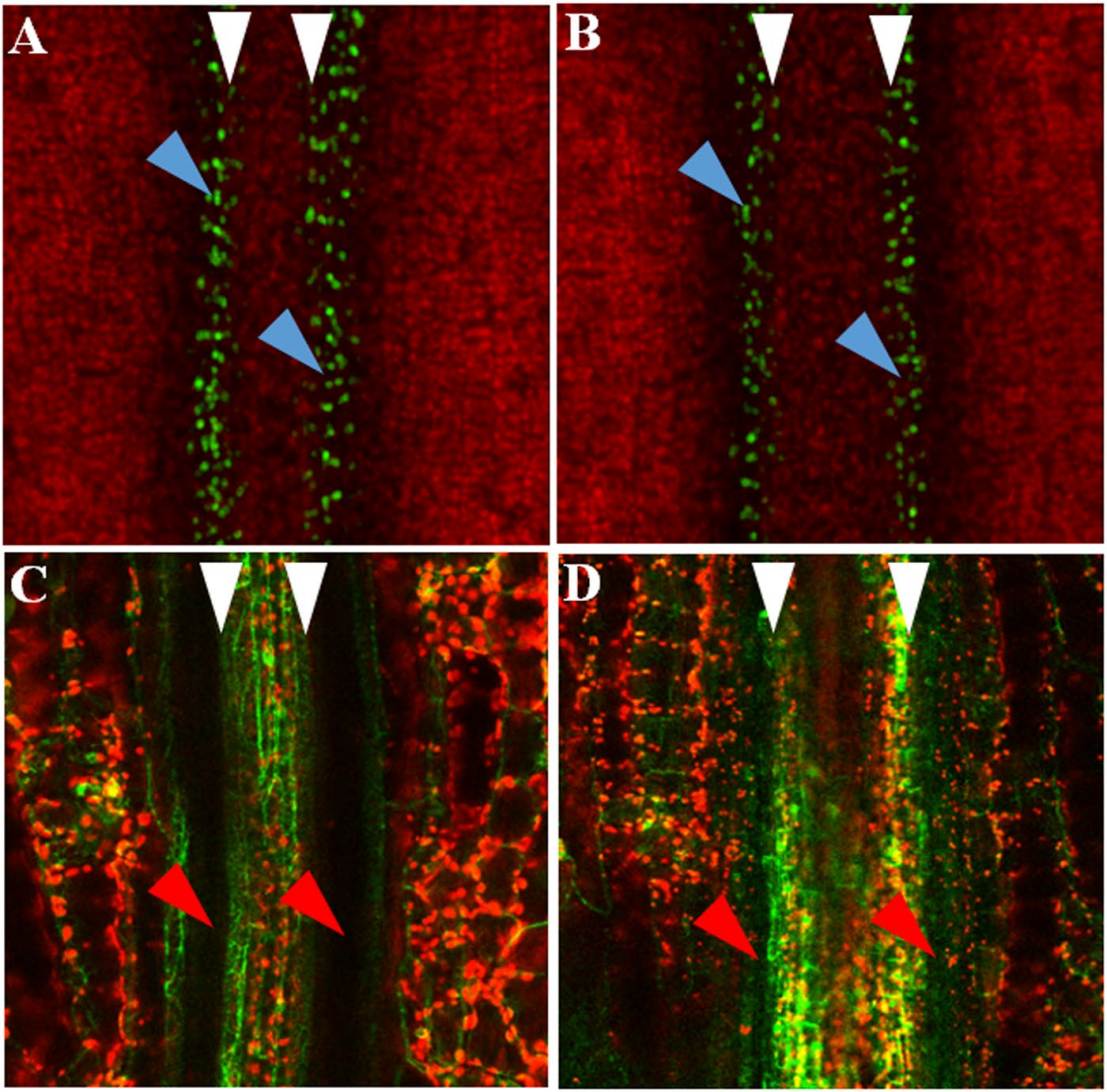
Visualized *AtIND* expression in valve margin and auxin signalling in Arabidopsis fruits. AtpIND-VENUS3x-NLS and DR5-GFP imaging in Arabidopsis fruits (in the valve margin at stage 17b) collected from plants treated with water (**A,C**) and Sealicit (**B,D**). Blue triangles indicate visible reduction of fluorescence intensity in Sealicit samples (**B**) in comparison to the respective water treated control (**A**) for AtpIND-VENUS3x-NLS. Red triangles represent activity of auxin responsive genes in the valve margin (**C**) and disrupted auxin activity in the medial section of the fruit collected from the plants treated with Sealicit (**D**). White triangles indicate increased size of the replum in Sealicit treated fruits (**B**,**D**). Experiments were performed in triplicate (n = 3).

It has been reported that fully indehiscent siliques (*ind-2* mutant) display a lack of differentiated valve margin and a wider replum due to the ectopic expression and activity of AtFUL and AtRPL transcription factors^12,13^. In order to assess the Sealicit MOA and its impact on dehiscence zone formation, we tested the expression levels of genes that determine valve (*AtFUL*)^20^ and replum (*AtRPL*)^19^ development, as well as a key player involved in valve margin formation (*INDEHISCENT*; *AtIND*). Real-time quantitative PCR (RT-qPCR) of these genes was conducted in reference to three house-keeping genes *ACTIN8* (*ACT8*)^45^, *GLYCERALDEHYDE-3-PHOSPHATE DEHYDROGENASE* (*AtGDPDH*)^46^, *UBIQUITIN CONJUGATING ENZYME E2 21* (*AtUBC21*)^47^ (for primers see Table S1). Ubiquitin-conjugating enzyme *AtUBC21* was found to be the most consistently expressed house-keeping gene, hence was used for normalisation. Sealicit was found to significantly decrease *AtIND* (*p* ≤ 0.001) and *AtFUL* (*p* ≤ 0.001) expression and simultaneously upregulate *AtRPL* (*p* = 0.31) (Fig. 2A). These results could account for alterations in fruit morphology and reduced silique opening. To better understand *AtIND* expression, dynamics confocal microscope was used to image the transcriptional fusion AtpIND-3xVENUS^48^, a reporter of *AtIND* promoter activity. Imaging of fruits at stage 17b revealed not only a noticeable decrease in the fluorescence level but also a wider replum (Figs 3B and S2B).

### Sealicit effects are concentration dependent

The final outcome of any plant biostimulant treatment is determined by the applied dose. In order to test whether the effect of Sealicit is concentration dependent, the dose of applied biostimulant was halved and doubled. We observed that all Sealicit concentrations significantly (*p* = 0.029) reduced *AtIND* expression (Fig. 2B), from the highest to lowest respectively. Subsequently, to test if there was a correlation between Sealicit concentration, *AtIND* expression and pod firmness, RIT testing was performed. Interestingly, the lowest Sealicit concentration reduced fruit opening, while the highest concentration had a smaller effect (Fig. S1C), this demonstrates that the physiological and morphological effects are concentration dependent, but not in a linear manner.

### Sealicit modulates auxin-mediated fruit development

Plant biostimulants have been shown to have a positive effect on plant growth, which in part has to do with plant signalling interference^49^. Macroalgal derived oligosaccharides have been demonstrated to influence auxin biosynthesis^36^ and distribution in the roots of rice, improving root and shoot growth^50^. To test this hypothesis, an auxin signalling reporter line DR5-GFP in Arabidopsis fruits (stage 17b) was analysed. Plants sprayed with Sealicit showed considerably altered fluorescence signal intensity when compared to the control (Fig. 3D,C, respectively, and Fig. S2A). This physiological change could account for disrupted auxin levels within the valve margin. Similarly to the transgenic Arabidopsis AtpINDx3VENUS-NLS^48^, imaging of fruits treated with Sealicit at stage 17b also revealed a wider replum (Fig. 3D).

### Sealicit effect on pod morphology in winter OSR varieties

The Brassicaceae family comprises of approximately 3700 species that typically develop dry dehiscent fruit^5^. In order to test whether the Sealicit effects observed in Arabidopsis could be repeated in another member of the Brassiceae tribe that develops elongated and tubular siliques^5^, a similar set of experiments in six commercial winter OSR varieties (WOSR) were performed (see V1-V6, Table S1).

**Table 1:** WOSRs commercial varieties used for field trials and assessment of pod shattering.

Variety name	Conventional/hybrid	Recommended 2017	Recommended 2018	Description
V1	Hybrid	AHDB	AHDB	High yield
V2	Hybrid	DAFM	DAFM	High yield Pod shatter resistant
V3	Conventional			High yield
V4	Hybrid	DAFM, AHDB	DAFM, AHDB	High yield
V5	Hybrid		AHDB	High yield Pod shatter resistant
V6	Conventional	DAFM, AHDB	DAFM	High yield

Pod length and weight measurements revealed some significant differences between treated and control plants (Fig. S3). V1 and V4 displayed clear increases in both the length (V1 *p* ≤ 0.001; V4 *p* = 0.29) and weight (V1 *p* ≤ 0.001; V4 *p* ≤ 0.001), while V5 displayed the opposite effect (V5 length, *p* = 0.013; V5 weight, *p* ≤ 0.001) after Sealicit application. Treatments also showed a positive effect on pod weight in the case of the naturally shatter-resistant V2 (*p* ≤ 0.001), whereas V3 and V6 showed negative effects on this parameter (V3 *p* = 0.005; V6 *p* ≤ 0.001) (Fig. S3).

### Sealicit has a positive effect on WOSR pod shattering

**Figure 4:**
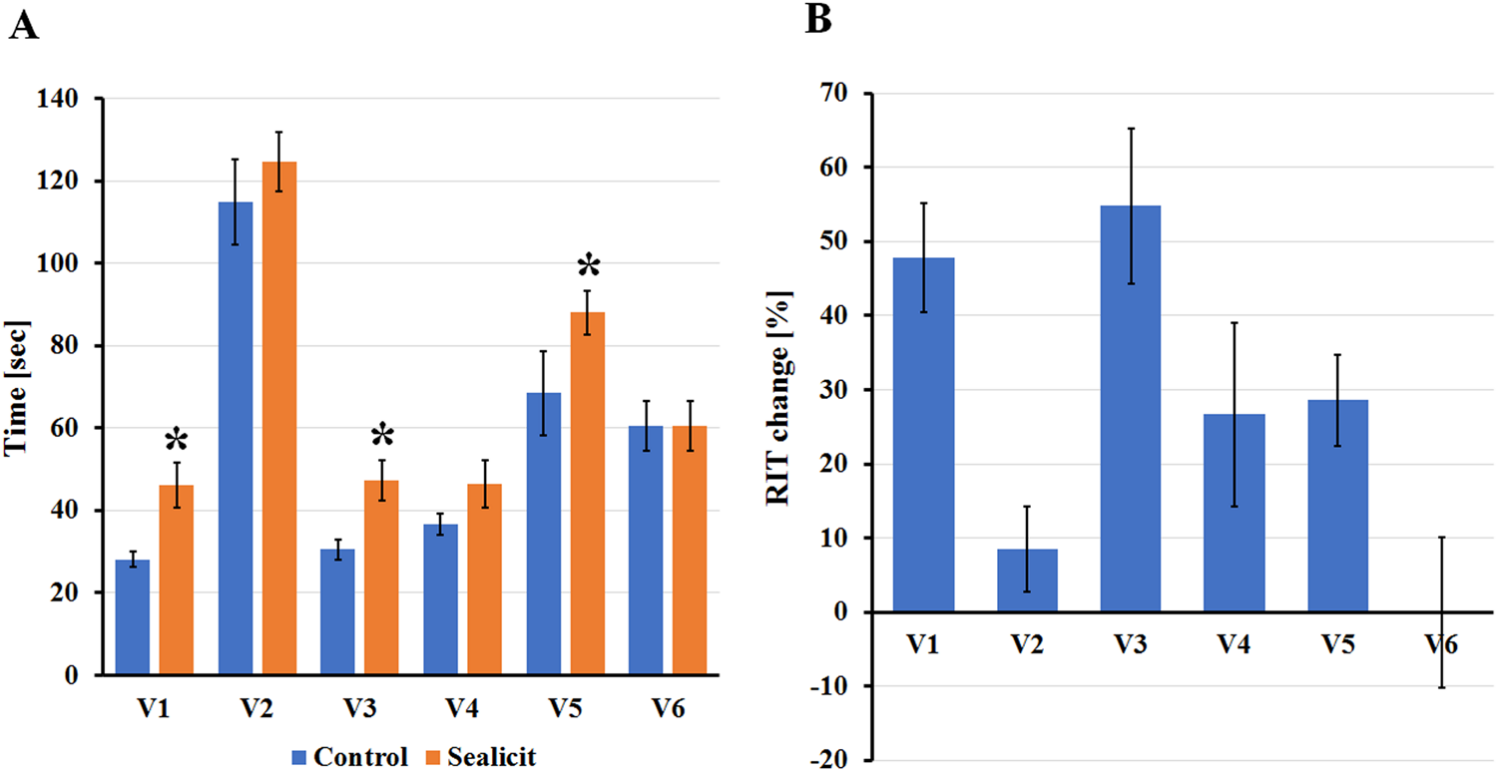
Firmness of WOSR pods tested using RIT. Chart on the left side represents pod firmness test by RIT on collected fruits from plants treated with water and Sealicit. Each bar represents the time at which 50% of pods were broken or damaged for six different WOSR varieties (V1-V6) (**A**). Chart on the right side represents relative breaking time change in treated pods of all tested WOSR varieties with respect to those from control plants (**B**). The error bars represent SE and means followed by asterisk within the same variety indicate significant differences between control and the Sealicit treatment based on t-test (*p* ≤ 0.05). Number of analysed samples (n = 60).

For quantifying and comparing the exact pod shattering potential between individual varieties, the RIT was performed on dry pods at BBCH 99^44^. Among all tested WOSR varieties, V2 and V5 were the most pod shatter resistant when compared to the others (Fig. 4). These results were coherent with the fact that these commercial genotypes are described as hybrids resistant to pod shattering. This observation indicated a good experimental design, as well as the robustness and sensitivity of the RIT method. Moreover, pods of Sealicit treated plants showed a different shatter time in comparison to those obtained from water sprayed control (Fig. 4). Sealicit significantly increased pod firmness of all varieties (V1 *p* ≤ 0.001; V3 *p* = 0.003; V5 *p* ≤ 0.001), except V4 and V6, which did not show any significant change. These results indicated that Sealicit was able to reduce pod shattering, however the size of the effect was variety dependent.

### Sealicit is elevating total lignin content in replum of WOSR pods

Kuai *et al*., 2016 reported that increased lignification in the whole pod correlates with improved pod shattering in different OSR varieties^51^. Using a similar approach, the effect of Sealicit on the total lignification of the replum was measured. Interestingly, pods collected from Sealicit treated plants produced increased amounts of lignin in each WOSR variety tested. The largest increases were in varieties V4 (*p* = 0.008) and V5 (*p* = 0.009) (Fig. S4).

### *BnIND* expression analysis

**Figure 5:**
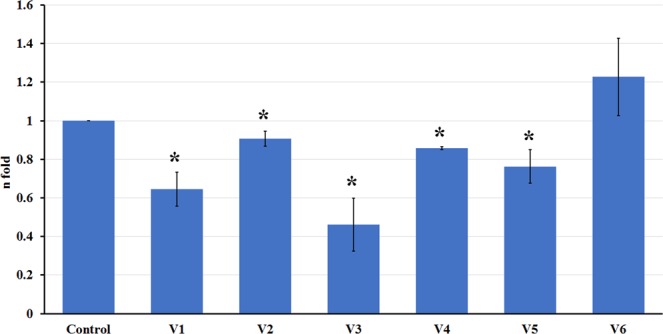
Relative *BnIND* expression in varieties treated with Sealicit. Results are displayed as the relative log2 fold-change with respect to the *BnEF1a* gene expression levels. The error bars represent SE and means followed by asterisk indicate significant differences between control and the Sealicit treatment based on one-way analysis of variance (ANOVA). The significance level was set at *p* ≤ 0.05 and performed by Holm-Sidak’s test. RNA extractions followed by RTq-PCR experiments were performed in triplicate (n = 3).

The transcript levels of *BnIND* were determined to establish if the expression levels were related to reduced pod shatter. Transcript levels were determined using RT-qPCR in different WOSR varieties in reference to three house-keeping genes: *BnACTIN2* (*BnACT2*)^45^, *UBIQUITIN11* (*BnUBQ11*)^52^ and, *TRANSLATION ELONGATION FACTOR1* (*BnEF1a*)^53^. The most consistently expressed house keeping gene was *BnEF1a*, hence this was used for normalisation of expression levels (for primers see Table S2). Sealicit was found to significantly decrease *BnIND* expression in all tested varieties (V1 *p* = 0.016; V2 *p* = 0.010; V3 *p* = 0.017; V4 *p* ≤ 0.001; V5 *p* = 0.050) except V6 (Fig. 5), which is in agreement with the RIT analysis.

### WOSR field trials and yield

**Figure 6:**
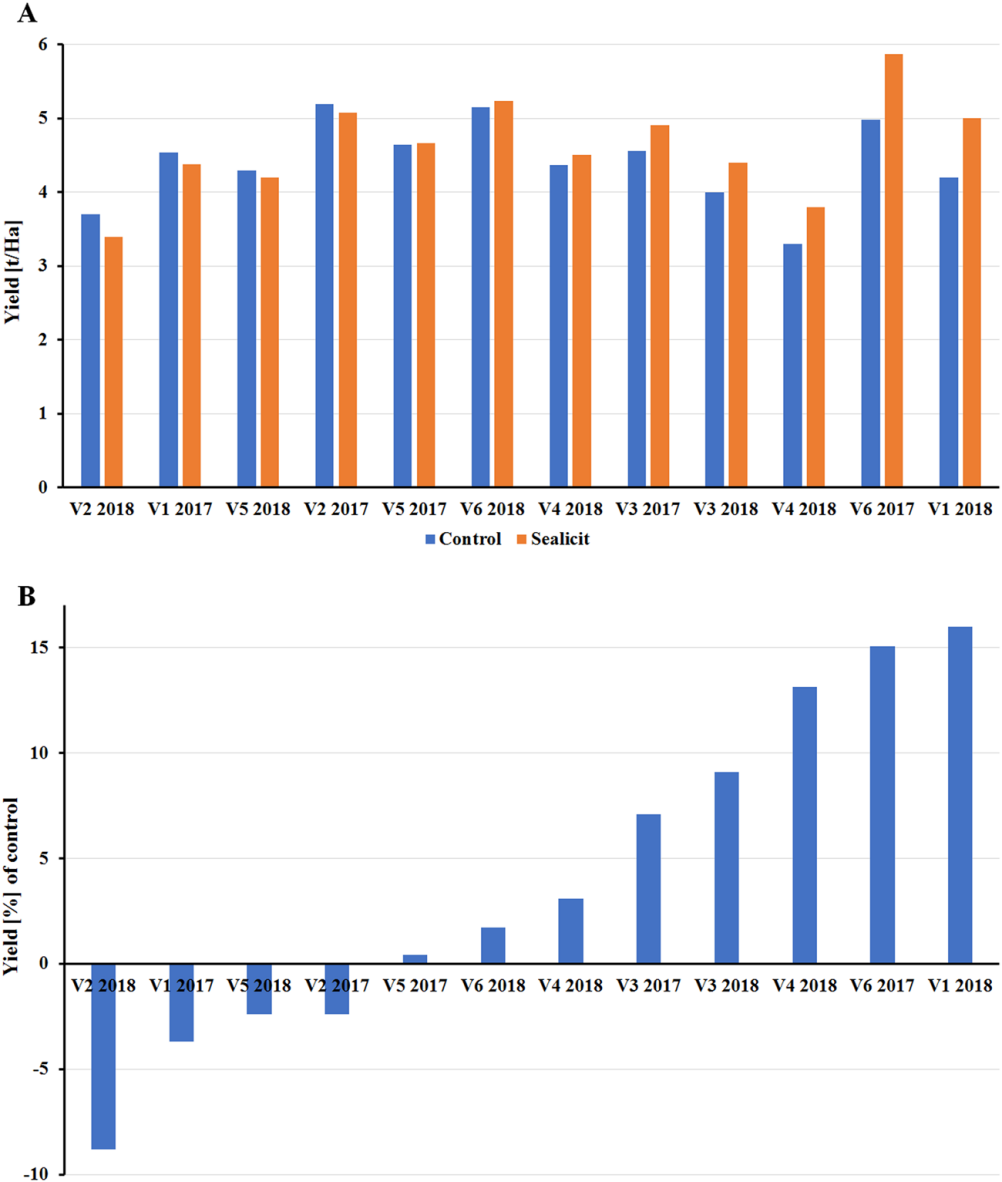
Seed yield evaluation recorded for winter varieties treated with Sealicit in seasons 2016/2017–2017/2018. Charts represent total seed yield per hectare (**A**) and yield uplift with respect to control (**B**) across two seasons for six recommended WOSR varieties. One plot per WOSR variety and season was evaluated for WOSR plants treated with water and Sealicit.

Evaluation of the impact of Sealicit application on yield in WOSR varieties in field conditions, involved performing open-field trials in county Cork in Ireland in two consecutive seasons (2017–2018). All WOSR varieties mentioned above were sprayed with the appropriate concentration of Sealicit at 6–8 leaf stage (BBCH 16–18^54^), grown for 45–48 weeks and sprayed with a desiccant three weeks prior the harvest. Tested WOSR varieties showed fluctuations on harvested seed yield in their response to Sealicit, which could be additionally influenced by the type of soil and changing weather conditions (Fig. 6A). On average, Sealicit treated varieties produced a yield increase of 4.5%. The largest negative yield change was observed for V2 (−8.8%) and the largest increase for V1 (16.0%) (Fig. 6B). Interestingly, Sealicit showed increased yield for all varieties except for the shatter resistant hybrids V2 and V5 (Fig. 6A).

## Discussion

The demand for increased agricultural production in the context of arable land scarcity and climate change means that new agricultural solutions are required to meet these challenges^1^. In the last decade, plant biostimulants have gained significant attention for their ability to enhance crop productivity^31^. In our study, we have evaluated the MOA of Sealicit which was developed to tackle one of the major agricultural traits impacting on the productivity of OSR (pod shattering).

### MOA of Sealicit

In parallel to a detailed analysis of the effect of Sealicit on a closely related model plant to OSR (*Arabidopsis thaliana*), we tested whether similar effects can be observed in field conditions in commercial WOSR varieties. The impact of Sealicit on fruit/pod shattering was assessed by diverse approaches; (i) pod morphology; (ii) testing pod shattering by performing RIT; (iii) analysing the expression level of the major transcription factors determining pod development and shattering; (iv) measuring OSR replum lignification; and (v) OSR seed yield assessment at harvest. Morphological analysis indicated that Sealicit MOA affected the biomass. However, while a significant increase in fruit size was observed in Arabidopsis fruits (Fig. S1), this effect was not consistently found in all the WOSR varieties tested (Fig. S3B), therefore the seed yield increase was related to other pod features. On the other hand, Sealicit had an impact on replum expansion and valve margin development in Arabidopsis fruits, which could account for the improved indehiscence. Pod firmness tests (RIT), showed significant improvement in Arabidopsis and in treated OSR, but with a certain degree of variability. This variability in reducing shatter can be partially explained by variety/genotype specific responses to Sealicit.

Sealicit treated Arabidopsis and five out of the six tested WOSR varieties showed a correlation between the extended RIT half-life time and decreased *IND* expression levels (Figs 1, 5 and S1C). The variety V6 that did not show the correlation, however still produced a higher yield in comparison to the control. On the other hand, V2 which showed a weak correlation between RIT and *BnIND* expression and produced a lower seed yield when it was sprayed with Sealicit. This result could be linked to the low *BnIND* levels measured in this shatter resistant variety and indicate that further decreases of *BnIND* may have a negative impact on pod development and total seed yield, as low *IND* levels promote *FUL* and *RPL* ectopic expression^12,14,19,23^. Although, five varieties treated with Sealicit showed significant differences in *BnIND* expression levels, it has to be noted that the observed variability in the error bars for all samples may be partially due to single nucleotide polymorphism (SNP) present in *BnIND* gene sequences that may affect the primer binding and PCR reaction efficiency. Overall, the interesting correlation between pod firmness as tested by RIT, *IND* depletion and replum lignin amount suggests that Sealicit was not only able to improve pod shattering resistance, but also stimulate other pathways associated with lignin biosynthesis in the whole replum tissue.

Two seasons of field testing of Sealicit in commercially relevant WOSR varieties demonstrated its robustness and efficacy in improving seed yield. The average yield increase was recorded at 4.5% with a maximum of 16% (Fig. 6B) which translates to a good return of investment (ROI) per hectare. Considering that the application timing could be further optimized, the observed yield increase could be potentially higher. The negative effect of Sealicit on the yield of shatter resistant varieties is an interesting finding and indirectly validates the MOA (down regulation of *BnIND* expression) as decreasing the expression levels of V2 and V4 which are already low may lead to pods that are too firm with a resulting impact on yield. Overall, Sealicit improved pod firmness and decreased *IND* expression, these parameters were shown to correlate well with WOSR yield data. This indicates that part of the Sealicit MOA involves the modulation of IND-mediated dehiscence zone development, resulting in less pod shatter and higher seed yield.

### Sealicit reduces pod shattering through modulation of plant signalling

It has been previously reported that AtIND can regulate auxin flux and high auxin concentration can inhibit *AtIND* expression^27^, therefore we analysed the fluorescence signal intensity in the auxin signalling reporter line DR5-GFP in Arabidopsis fruits collected from Sealicit sprayed and control plants. Fruits from plants treated with Sealicit showed a disrupted auxin signalling at stage 17b which may suggest that the MOA of Sealicit encompasses the local modulation of auxin homeostasis in the dehiscence zone, interfering with its development and further fruit growth^27^. Additionally, this effect coincided with a replum widening in the auxin signalling reporter and the transgenic Arabidopsis AtpINDx3VENUS-NLS^48^, which has also been reported as a direct effect of decreased *AtIND* in *ind* knockouts or elevated *AtRPL* gene expression^12,19^. Therefore, our confocal microscopy results were in agreement with the obtained qPCR expression results for *AtIND* and *AtRPL* and, consequently, they supported the observation that Sealicit affects the expression of both genes leading to altered fruit morphology and decreased pod shatter (Figs 2 and 3). Taking into account that *AtRPL* and *AtBP* are transcription factors that can also interact with the auxin response factor ARF3/ETTIN^55^, it is plausible to speculate that Sealicit is promoting endogenous auxin biosynthesis and/or modifying auxin efflux out of the valve margin, which leads to the formation of shatter resistant pods. This hypothesis could be tested by analysing the induced expression of auxin biosynthesis (TAA1/TAR and YUCCA families)^56,57^, transporter genes (PIN3) or phosphorylation-based PIN recruiting machinery (PINOID, WAG2)^27,28^ in the fruits of plants treated with Sealicit. Another possibility would be to test the effect of Sealicit on their respective genetic mutants. There is also increasing evidence that intracellular alterations in Ca^+2^ levels, can work as a secondary messenger during auxin-mediated signalling^58–60^. In fact, one of the key components of ANEs has previously been shown to influence calcium flux^50^. In order to gain a better understanding of Sealicit MOA it would be interesting to further explore auxin dynamics in relation to Ca^+2^ release, perception and Ca^+2^ responsive proteins in the context of fruit development and pod shattering.

## Summary and Perspectives

Plant biostimulants and in particular ANEs are emerging as effective crop inputs that improve plant growth and health, mitigate effects of stressful conditions, improve crop quality and, most importantly, increase yield. Although there are many reports on diverse biostimulant formulations and their efficacy in numerous plant systems, the identity of active molecules, their perception and downstream signalling are still poorly documented. This work shows a direct connection between OSR treatment with a specific biostimulant formulation, genetics and plant physiology underpinning the seed dispersal mechanism, which consists a significant leap forward in understanding a biostimulant’s mode of action.

Confirming the initial hypothesis, our results showed that the biostimulant Sealicit affected the development of the dehiscence zone by disrupting the expression of a crucial genetic player in pod shatter (*BnIND*). Our data show that Sealicit improves pod firmness thereby reducing pod shattering and could therefore be an attractive solution for efficient OSR production. The methods utilized here could easily be used in other economically important podded crops. For example, soybean could be an interesting target because it is a member of the Brassicaceae family that employs a similar strategy of seed dispersal for successful reproduction. Although, pod shattering depends on the variety and weather conditions, plant biostimulants such as Sealicit have the potential to activate the crops genetic potential to achieve a high quality crop with enhanced yield omitting time consuming, labour intense breeding and still controversial genetic modifications. ANE biostimulants like Sealicit, that are robust, effective and scientifically validated, offer entirely new solutions for tackling specific developmental aspects or crop traits that can be concealed, like in the case of pod shattering, or apparent such as increased fruit set, size, uniformity or longer shelf life.

## Material and Methods

### Plant material and growth conditions

*Arabidopsis thaliana* ecotype Col-0, AtIND-3xVENUS-NLS^48^ and auxin signalling reporter line DR5-GFP^61^ were grown in a growth cabinet at 22 °C in long day conditions (16 h of light and 8 h dark) under light intensity of 100 µmol m^−2^s^−1^ and 80% relative humidity. Six commercial WOSR varieties (V1-V6) were tested in variable weather conditions through open-field trials. All the WOSR seeds were kindly provided by farmers. WOSR varieties were selected according to the recommendations of the Irish Department of Agriculture, Food and the Marine (DAFM) and the British Agriculture and Horticulture Development Board (AHDB) as the putatively highest yielding varieties in the seasons 2016–2018 (Table 1). Sealicit was foliar sprayed at 6–8 leaf stage, which corresponds to the stage 16–18 according to the BBCH scale. This system used for uniform coding for phonologically similar growth stages of all mono- and dicotyledonous plant species^54,62^. Samples for morphology, lignin content, gene expression, RIT analysis and yield assessment were collected between the stages 17b and 19 in arabidopsis fruit (BBCH 71–75 and 99 in OSR). Fully senescent plants were sprayed with a desiccant three weeks prior to the harvest.

### Treatments application

0.5 mL of commercial water-soluble Sealicit (containing PSI-759 complex, Brandon Bioscience, Tralee, Ireland) solutions were applied by single foliar spray at a dilution of 1/1200; 1/600; 1/300 to 4 to 5-week-old Arabidopsis plants (fully developed rosettes and bolting plants with developed auxiliary branches and first flower buds). Sealicit was applied on WOSR by single foliar spray at a dilution of 1.5 L/Ha at BBCH 16–18. Control plants were sprayed with equal volume of distilled water.

### Experimental design of field trials

The open-field trials with six WOSR varieties were laid out in a randomized block design with single plots in two consecutive years from September 6, 2016 to July 25, 2018 at Shanagarry, County Cork, Ireland. The unit plot size for each experiment was 12 m × 4 m and buffer distances between plots were 0.2 m. The soil type was medium loam over limestone with a pH of 7.5 and soil nutrition tests showed sufficient amount of phosphorous (soil at index 4) and medium levels of magnesium, potassium, copper, manganese and zinc (soil at index 3). Previous crop was barley and soil N index was 2^63^. For optimal plant growth and development, recommended fertilizer, fungicide, insecticide, herbicide and desiccation rates were applied. All agricultural practices and pest control measures were applied according to the Agriculture and Food Development Authority (Teagasc) recommendations^64^. The density of plants per square meter was 35 and 55 for hybrids and conventional varieties respectively. The plots were harvested at BBCH 99 using a combine harvester and seed yield was recorded. Before harvesting, fifteen plants were picked randomly from each plot and pods were sampled to determine the RIT and phenotypic traits.

### Random impact test (RIT)

RIT of dried OSR pods was performed according the protocol developed and described by Arnaud *et al*.^44^. In principle, six randomly picked oilseed rape pods at BBCH 99 were mixed with 15 metal beads (13 mm in diameter each) and shaken for 11 seconds until half of the pods were broken or damaged. The number of cycles was recorded and the pod shattering resistance was expressed as the half-time of each sample. Regarding Arabidopsis fruits, 10 siliques at stage 18 (yellow, fully ripen but yet perfectly intact) of each sample were collected and placed with 3 mm metal beads on a 30 mm diameter petri dish installed on the top of a vortex mixer. After shaking them for 30 seconds at speed 3 inside a Petri dish (30 mm diameter) fixed on the top of the vortex, the percentage of open or damaged fruits was recorded to calculate the difference in shattering. All OSR and Arabidopsis varieties sprayed with water or Sealicit were tested at least 40 times.

### Pod phenotypic traits

Fully developed Arabidopsis fruits at stage 17b, according to classification of Smyth *et al*.^43^, and dried OSR pods used for the RIT test described above were measured using ImageJ and weighed.

### Confocal microscopy

Gynoecia at stage 17b of transgenic Arabidopsis AtpINDx3VENUS-NLS^48^ and auxin signalling reporter line DR5::GFP^61^ were imaged using Laser Scanning Microscopy Leica SP8. Fluorescence signal of AtIND-VENUS3x-NLS was measured using segmented line drawn in the middle of valve margin o respective fruits. Fluorescence of DR5-GFP was registered by measuring signal intensity per area and normalizing the signal between measured boxes by dividing the signal intensity by size of measured area. Abovementioned measurements were performed using the latest version of Fiji (ImageJ). Obtained values were subsequently used to generate Box & Whisker charts (Fig. S2).

### RNA isolation and qRT-PCR

RNA was obtained from Arabidopsis fruits and OSR pods (at stage 17b which corresponds to BBCH 71–75). Expression analysis were performed by real time-PCR using a Roche LightCycler 96 System (Roche, UK). Quantitative PCR was performed using the LightCycler RNA Master SYBR Green I one-step kit (Roche, UK) according to the manufacturer’s instructions. The expression level of genes of interest was expressed in n-fold change and calculated according 2^−ΔΔCT^ ^65^. Sequences of the specific primers are shown in Tables S1 and S2.

### Lignin determination for OSR replum pods

The quantification of lignin from replum of OSR pods at BBCH stage 99 was assessed according to the acetyl bromide method described by Barnes and Anderson^66^.

### Statistical analysis

Statistics were evaluated with the SigmaPlot 12.0 software for Windows. When making single or multiple statistical comparisons to test for significant differences in experiments, one sample t-test or one-way analysis of variance (ANOVA) were used, respectively. The normality and equal variance assumptions were checked. The significance level for both parametric tests was set at *p* ≤ 0.05 and the post-hoc analysis from ANOVA were conducted using the Holm-Sidak’s test. All data except the Arabidopsis fluorescence measurements (Fig. S2) and OSR yield data (Fig. 6) were reported as mean ± standard error (SE).

### Summary

A plant biostimulant produced from the brown seaweed *Ascophyllum nodosum* (Sealicit) when applied to oilseed rape (OSR) leads to disrupted auxin signalling in the valve margin, downregulation of *IND* expression and increased pod firmness. These changes stimulated a reduced pod shattering and thereby more harvestable seed yield in a number of varieties of OSR.

## Supplementary information

Supplementary Information
