# Supplementary material for: A plant biostimulant from the seaweed Ascophyllum nodosum (Sealicit) reduces podshatter and yield loss in oilseed rape through modulation of IND expression
Source: Sci Rep. 2019 Nov 12;9:16644. doi: 10.1038/s41598-019-52958-0 (PMC6851122; doi:10.1038/s41598-019-52958-0)
Supplement: Supplementary file 1 — Supplementary Information [file 41598_2019_52958_MOESM1_ESM.pdf]

A plant biostimulant from the seaweed Ascophyllum nodosum (Sealicit) reduces pod-shatter and yield loss in oilseed rape through modulation of *IND* expression

Łukasz Łangowski[b], Oscar Goñi[a], Patrick Quille[a], Pauline Stephenson[c], Nicholas Carmody[b], Ewan Feeney[b], David Barton[b], Lars Østergaard[c], Shane O'Connell[a]*

[a] Plant Biostimulant Group, Shannon Applied Biotechnology Centre, Institute of Technology Tralee, Clash, Tralee, Co. Kerry, Ireland

[b] Brandon Bioscience, Centrepoint, Tralee, Co. Kerry, Ireland

[c] Department of Crop Genetics, John Innes Centre, Norwich Research Park, Colney, NR4 7UH Norfolk, Norwich, United Kingdom

SUPPLEMENTARY MATERIALS

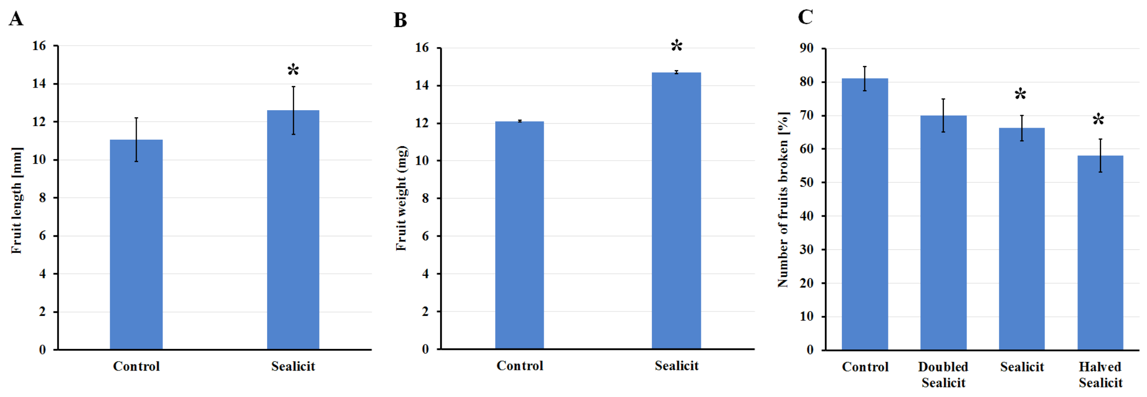

Supplementary Figure 1. Arabidopsis fruit phenotype and firmness assessment.

Charts represent Arabidopsis fruit length (A), weight (B), and fruit firmness tested by RIT on fruits collected from plants treated using three different concentrations of Sealicit (C). The error bars represent SE. The fruit length and weight means followed by asterisk indicate significant differences between control and the Sealicit treatment based on t-test ($p \leq 0.05$). Fruit firmness means followed by asterisk indicate significant differences between control and the Sealicit treatment based on one-way analysis of variance (ANOVA). The significance level was set at $p \leq 0.05$ and performed by Holm-Sidak's test. *p* (Doubled Sealicit) = 0.097; *p* (Standard Sealicit) = 0.026; *p* (Halved Sealicit) = 0.003. Number of analysed samples (n = 40).

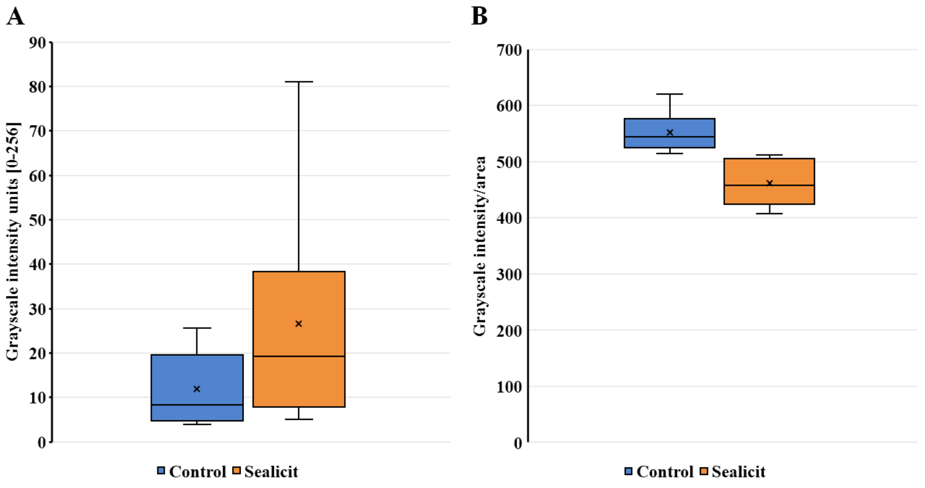

Supplementary Figure 2. Relative fluorescence change between control and samples treated with Sealicit.

Box & Whisker charts represent fluorescence increase of AtpIND-VENUS3x-NLS (A) and fluorescence decrease of DR5-GFP (B) in Arabidopsis fruits (in the valve margin at stage 17b), collected from control plants and treated with Sealicit. Fluorescence signal was measured along both valve margins by triplicate (n=3). Error bars represent minimal and maximal signal registered, horizontal line represent the median of signal registered. The x markers represent mean value of all measured signals.

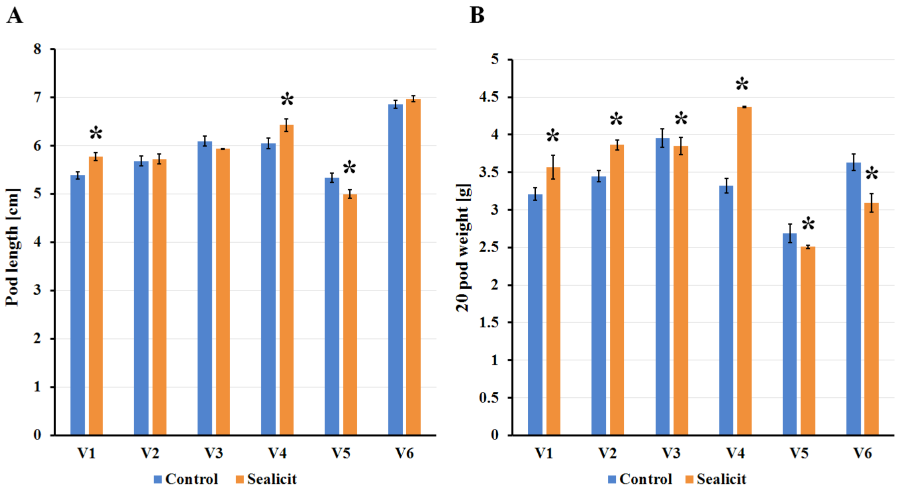

Supplementary Figure 3. Pod length and weight in OSR winter varieties treated with Sealicit.

Charts represent OSR single pod length (A) and 20 pods weight (B) of dried pods collected from WOSR plants treated with water and Sealicit. The error bars represent SE and means followed by asterisk within the same variety indicate significant differences between control and the Sealicit™ treatment based on t-test ($p \leq 0.05$). Number of analysed samples (n = 60).

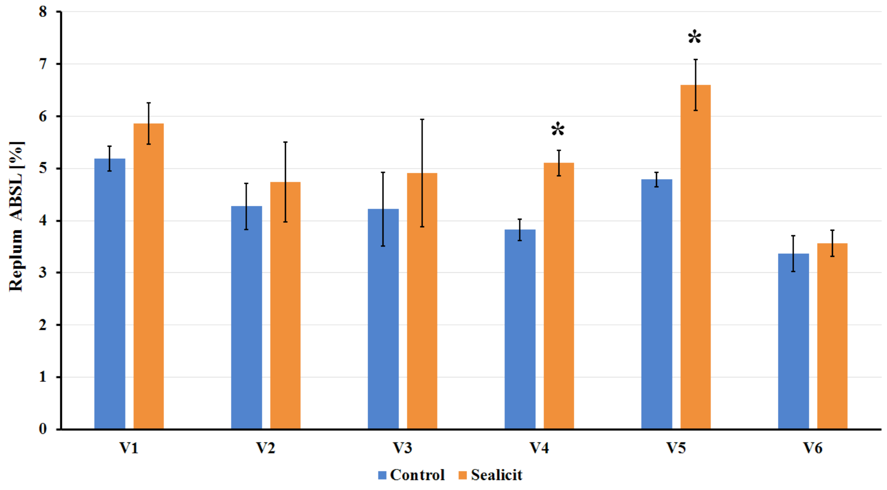

Supplementary Figure 4. Replum lignification of winter OSR pods treated with Sealicit. Charts represent replum lignification on pods expressed as %ABSL in the replum's alcohol insoluble residue. The error bars represent SE and means followed by asterisk within the same variety indicate significant differences between control and the Sealicit treatment based on t-test ($p \leq 0.05$). Number of analysed samples (n = 60).

Table S1. Primers used for RT-qPCR analysis in Arabidopsis.

AtIND_FW, AT3G58780	GAAAGGATCAGCGAGAAGAT
AtIND_REV, AT3G58780	CTGCCGTTTCAAGAACTTG
AtUBC21_FW, AT5G25760	GTCCTCTCAACTGCGACTC
AtUBC21_REV, AT5G25760	GTGTGTACATGCGTGCCAT
AtGAPDH_FW, AT1G13440	GAATCGGTCGTTTGGTGG
AtGAPDH_REV, AT1G13440	GTCATGTACTCGGTGGTGA
AtACT8_FW, AT1G49240	CATCATGGTGTCATGGTTG
AtACT8_REV, AT1G49240	CAGGAGCAATACGGAGCT
AtFUL_FW, AT5G60910	GGCAAACTCTTCGAATATTC
AtFUL_REV, AT5G60910	GTACCTCAACTCTTGCCTTG
AtRPL_FW, AT5G02030	GTTCTAAGGGCTTGGCTCT
AtRPL_REV, AT5G02030	GCGTTTATGAACCAATTCGA

Table S2. Primers used for RT-qPCR expression analysis in *Brassica napus*.

BnIND_FW, BnaA03g27180D	GAAAGGATAAGCGAGAAGAT
BnIND_REV, BnaA03g27180D	CTGCCGTTTCAAGAACTTG
BnEF1a_FW, BnaC08g17710D	GTAGAGAAAGCTGGTATATGGC
BnEF1a_REV, BnaC08g17710D	GCTCCACTAATCATATTGGGTAC
BnUBQ_11_FW, BnaC04g09440D	GATTACAACATCCAGAAGGAG
BnUBQ_11_REV, BnaC04g09440D	GGATCCCTTCCTTGTCTTG
BnACT2_FW, BnaA05g21350D	GATCTGGCATCACACTTTC
BnACT2_REV, BnaA05g21350D	CCAGAATCCAGCACAATAC
